# Small Lymphocytic Lymphoma Presenting as a Breast Lump: A Rare Presentation of Non-Hodgkin's Lymphoma

**DOI:** 10.7759/cureus.19401

**Published:** 2021-11-09

**Authors:** Nazia Khan, Hamid Shaaban, Gunwant Guron

**Affiliations:** 1 Oncology, St. Mary's Medical Center, Passaic, USA; 2 Hematology/Oncology, St. Michael's Medical Center, Newark, USA

**Keywords:** chornic lymphocytic leukemia, breast mass, small lymphocytic lymphoma

## Abstract

Approximately one-fourth of non-Hodgkin's lymphomas (NHLs) present with an extranodal origin. Primary and secondary involvements of the breast by lymphoma are rare because of the paucity of lymphoid tissue in the breast. Primary breast small lymphocytic lymphoma (SLL) typically presents as a manifestation of widespread chronic lymphocytic leukemia (CLL). A 58-year-old female presented to our clinic with a palpable breast mass. She had no cytopenias and her absolute peripheral B lymphocyte count was less than 5000/microL. The mass was biopsied and histology with immunohistochemistry showed neoplastic cells positive for CD23 and CD5 confirming the diagnosis of small B cell lymphocytic lymphoma of the breast. Further imaging revealed extensive mediastinal and retroperitoneal lymphadenopathy. Histopathology of bone marrow biopsy revealed diffuse infiltration with SLL. The patient was treated with six cycles of fludarabine, cyclophosphamide and rituximab (FCR) with excellent clinical response. To our knowledge, this is the first case of SLL infiltration of the breast without CLL treated successfully with FCR.

## Introduction

Primary breast lymphoma is a rare disease entity. They comprise only 0.5% to 1% of non-Hodgkin’s lymphoma (NHL) and of breast malignancies, respectively. According to the literature, the most frequent were two types of B lymphomas: follicular lymphoma and diffuse large B-cell lymphoma [1]. Diagnosis is mainly made by pathology and immunohistochemistry. Imaging may be normal or mimic breast carcinoma [1-3]. Management differs from primary breast carcinoma in terms of lesser surgical excision and more role of rituximab-based chemotherapy. We present a unique case of primary small lymphocytic lymphoma of the breast with bone marrow involvement that was successfully treated with fludarabine, cyclophosphamide and rituximab.

## Case presentation

**Figure 1:**
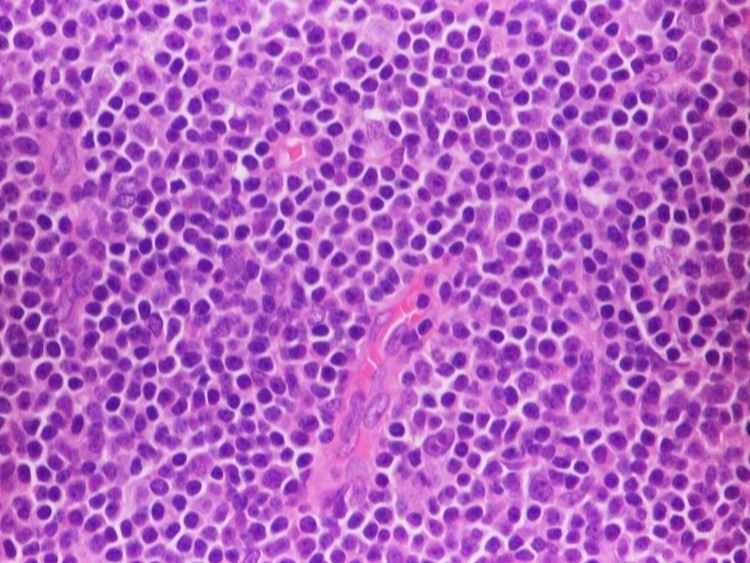
Histopathology of the breast biopsy showed diffuse atypical small lymphocytic cells.

**Figure 2:**
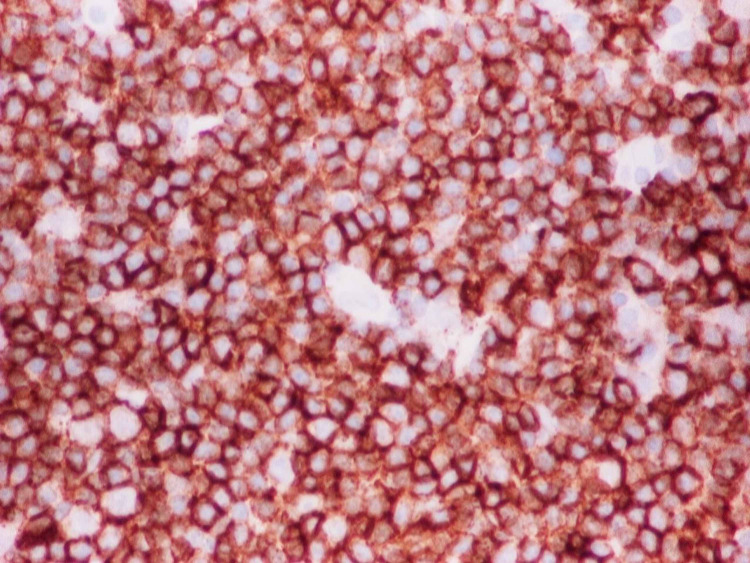
CD20 staining was positive.

**Figure 3:**
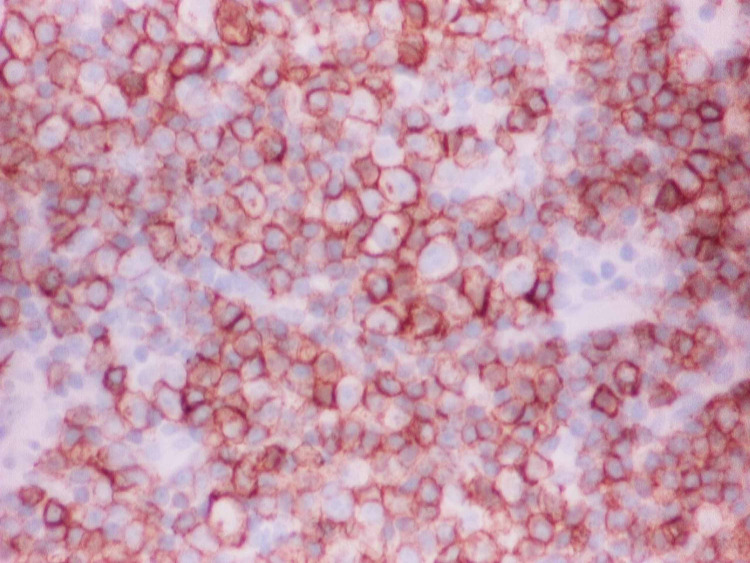
CD5 staining was positive.

**Figure 4:**
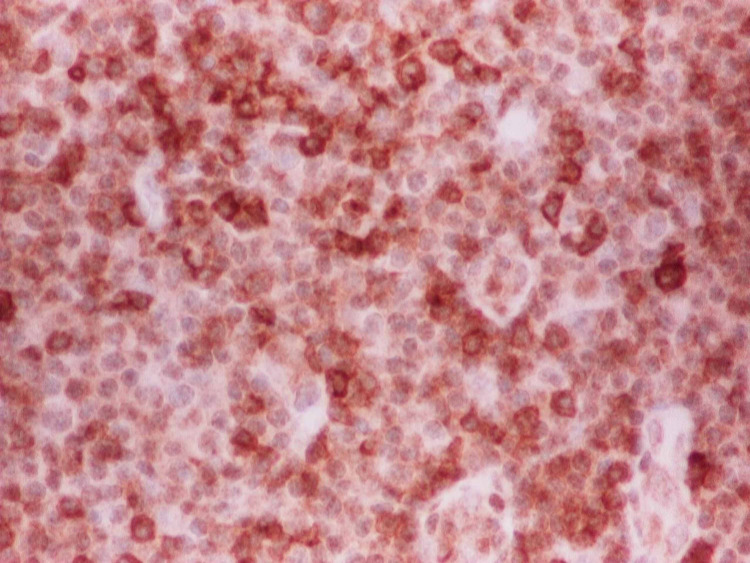
CD23 staining was positive.

**Figure 5:**
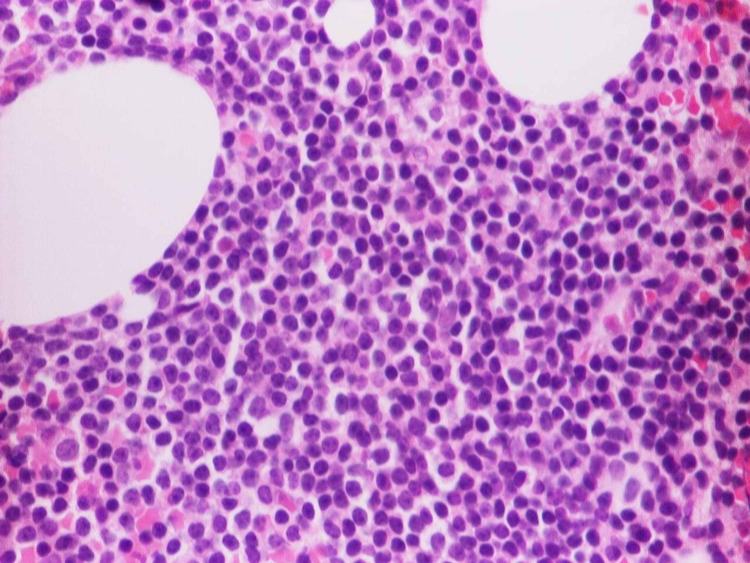
Bone marrow biopsy histopathology revealed diffuse involvement with small lymphocytic lymphoma.

**Figure 6:**
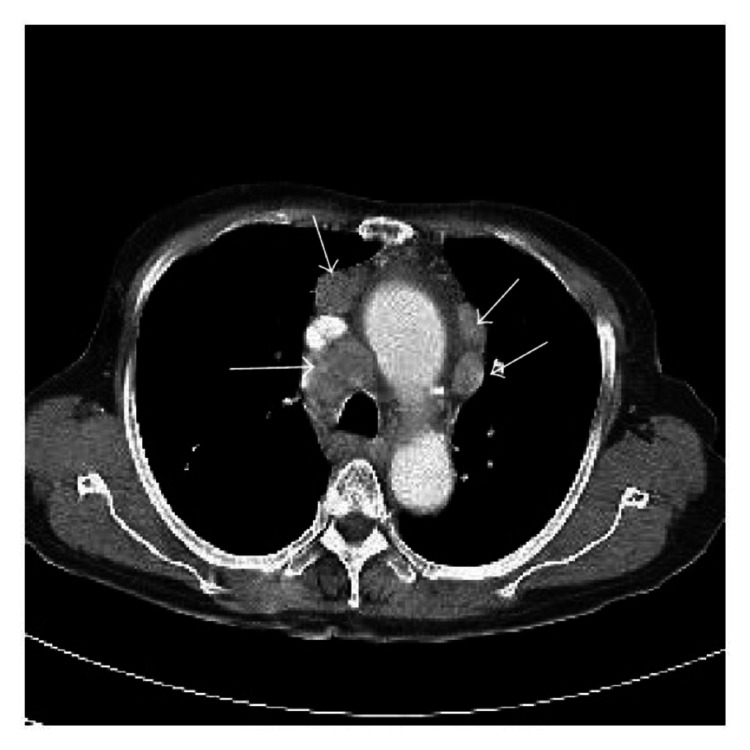
CT of the chest revealing mediastinal lymphadenopathy.

**Figure 7:**
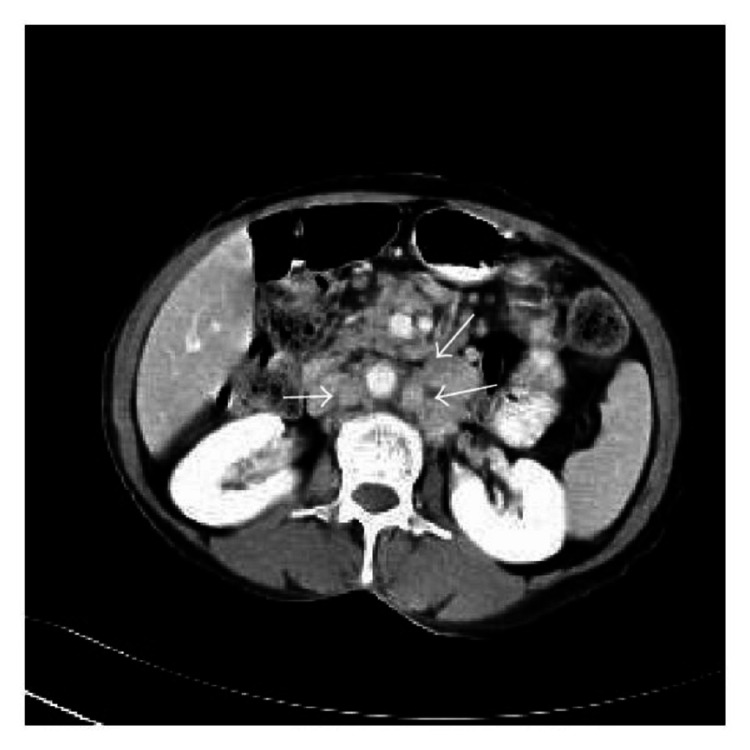
CT of abdomen revealing retroperitoneal lymphadenopathy.

A 58-year-old woman with a past medical history of diabetes and hypertension presented to our clinic for evaluation of an enlarging left breast mass that she first noticed three years ago. She stated that the mass started to progressively increase in size in the past few months. She also reported a weight loss of 34 pounds over the last nine months. On physical exam, she had a palpable breast mass of approximately 3 centimeters in the tail of the left breast. Rest of physical exam was unremarkable. Review of labs showed WBC of 8.8/mL with absolute lymphocyte count of 4400/mL, hemoglobin of 12g/dl and platelet count of 316K. Prior mammograms and ultrasounds had revealed stable intramammary lymph nodes at the site of the present lesion on the left breast over the last three years with no suspicious calcifications or architectural distortions. There were no palpable ipsilateral or contralateral axillary lymphadenopathy. The mass was subsequently percutaneously biopsied. Histopathology showed diffuse atypical small lymphocytic cells (Figure 1). Immunohistochemical staining revealed neoplastic lymphoma cells positive for CD20 (Figure 2), CD5 (Figure 3), CD23 (Figure 4), PAX5, CD4, BCL2 and negative for CD3, CD10, BCL1, and BCL6. The final pathologic diagnosis was consistent with primary small lymphocytic lymphoma of the breast rather than chronic lymphocytic leukemia. This was unexpected. Bone marrow biopsy was done and histopathology revealed diffuse involvement with small lymphocytic lymphoma (Figure 5). Fluorescence in situ hybridization (FISH) studies revealed trisomy 12 cytogenetic abnormality. Computed tomography (CT) imaging revealed extensive mediastinal lymphadenopathy (Figure 6) and retroperitoneal lymphadenopathy (Figure 7). The final clinicopathologic diagnosis was small lymphocytic lymphoma (Stage 4) with breast and bone marrow involvement. She was subsequently treated with the FCR regimen comprising of fludarabine, cyclophosphamide and rituximab which resulted in clinical and radiologic remission. The breast mass was no longer palpable and repeat CT scans did not reveal any evidence of any pathologic lymphadenopathy.

## Discussion

Small lymphocytic lymphoma (SLL) is a mature (peripheral) B cell neoplasm characterized by a progressive accumulation of functionally incompetent lymphocytes, which are monoclonal in origin. The neoplastic lymphoma cells identified in SLL and CLL have identical pathologic and immunophenotypic features; however, the combined absence of cytopenias and peripheral lymphocytosis > 5000/microL typically favors the diagnosis of SLL rather than CLL [4,5]. Less than 10% of patients with CLL present with isolated nodal involvement (i.e., SLL); this presentation accounts for less than 5% of all non-Hodgkin lymphomas. SLL is a neoplasm of monomorphic, small round B-lymphocytes, involving the peripheral blood, bone marrow, and lymph nodes [6]. In our case, it manifested as a breast tissue infiltrate with the morphology and immunophenotype of CLL with an insufficient number of circulating leukemia cells to fulfill the criteria for CLL. After extensive review of the medical literature, we could not identify any case reports of pure SLL of the breast without clinical CLL.

About one-fourth of patients with NHL present with an extranodal origin, which can arise from almost any organ in the body [3-5]. However, primary and secondary involvements of the breast by lymphoma are very rare occurrences. This is because of the paucity of lymphoid tissue in the breast. Non-epithelial breast neoplasms are usually lymphoid malignancies, with primary breast lymphomas (PBLs) representing less than 1% of malignancies of the breast. Most of the PBL are B-cell and T-cell lymphomas which uncommonly involve the breast [3-6]. The two most common lymphomas involving the breast are diffuse large B-cell lymphoma followed by extranodal marginal zone lymphoma of mucosa-associated lymphoid tissue (MALT lymphoma). B-lymphoblastic lymphoma, Burkitt's lymphoma, peripheral T-cell lymphoma, rarely, classic Hodgkin's lymphoma, and follicular lymphoma are other PBLs that are less frequently observed. Secondary breast lymphomas (SBL) are defined as the lymphomas with the breast being a minor site of dissemination and are most commonly the indolent follicular lymphoma [4-6]. Small lymphocytic lymphoma typically presents as a secondary manifestation of widespread CLL unlike our case in which leukemic cells were notably limited/absent. The usual presentation is a painless mobile mass not fixed to the chest wall or skin. CLL can involve the breasts diffusely and bilaterally, including involvement of the overlying skin [7]. Radiographic findings are generally non-specific and they may be mammographically occult as in our patient or rarely be associated with microcalcifications [4].

Diagnosis of SLL is mainly made by pathology demonstrating monoclonal small cells and immunostaining with confirmatory markers CD20, CD5 and CD23. Despite the fact that 50% of patients with B-SLL/CLL have abnormal karyotypes, none of them are diagnostic. It is interesting to note that trisomy 12 which was detected in our patient, is present in about 20% to 40% of the cases with cytogenetic abnormalities, and typically correlates with atypical histology, unusual clinical presentation and an aggressive clinical course [8-11]. Further clinical staging procedures including CT scans and bone marrow biopsies and aspirations can be done on a case by case basis. As was the case with our patient, at the time of diagnosis of SLL, about 90% of SLL patients have bone marrow involvement. There was evidence of generalized lymphadenopathy however there was notably an absence of circulating leukemia cells.

Management is often with rituximab-based chemotherapy in the front-line setting. Role of surgery is just limited to acquiring tissue for diagnosis and mastectomy offers no survival benefit. There are currently no up-to-date standard guidelines for the management and treatment of primary breast SLL. Mastectomy for any primary breast lymphoma is not well supported because it shows neither improved survival nor reduced risk of recurrence [9]. Also, axillary dissection is of no additional therapeutic benefit.

This is a very unusual case of small lymphocytic breast lymphoma in a woman with a painless mass in the left breast. The patient was successfully treated with FCR and achieved clinical remission. Even though it is very rare, it is important to consider this disease entity in the differential of a breast mass especially when it’s associated with indolent characteristics on imaging.

## Conclusions

Primary breast lymphoma (PBL) is a rare clinical entity accounting for 0.4%-0.5% of all breast neoplasms. SLL is a mature (peripheral) B cell neoplasm characterized by a progressive accumulation of functionally incompetent lymphocytes, which are monoclonal in origin. Primary breast small lymphocytic lymphoma usually presents as a manifestation of widespread Chronic Lymphocytic Leukemia. In our case, SLL manifested as a breast tissue infiltrates with the morphology and immunophenotype of CLL with an insufficient number of circulating leukemia cells to fulfill the criteria for CLL. This rare case highlights that the differential diagnosis for a painless breast mass should include pure SLL even in the absence of clinical CLL. These patients rarely require surgical resection and axillary lymph node dissection, and moreover, they respond clinically and radiologically to systemic anti-lymphoma therapy.
